# Identification of extracellular vesicle microRNAs as potential facilitators of interferon-alpha escape in Marek’s disease virus infection

**DOI:** 10.3389/fcimb.2026.1796248

**Published:** 2026-05-20

**Authors:** Shuang Wei, Zepeng Zhao, Jinping Dou, Weisong Gao, Xintao Gao, Tong Wu, Zhifang Zhang, Xingjian Liu, Yinü Li

**Affiliations:** 1 National Key Laboratory of Agricultural Microbiology, Biotechnology Research Institute, Chinese Academy of Agricultural Sciences, Beijing, China; 2 State Key Laboratory for Animal Disease Control and Prevention, College of Veterinary Medicine, Lanzhou University, Lanzhou, China

**Keywords:** extracellular vesicles, IFN-α, immune escape, MDV, microRNA

## Abstract

**Introduction:**

Marek’s disease virus (MDV) is a highly immunosuppressive alphaherpesvirus. However, whether and how MDV exploits extracellular vesicles (EVs) to evade host immunity, particularly the critical type I interferon (IFN-I) response, remains unknown. We hypothesized that MDV reprograms the EV microRNA (miRNA) cargo to facilitate its escape from the IFN-I-mediated antiviral state.

**Methods:**

Small RNA (sRNA) sequencing was conducted to profile and compare the expression patterns of EV miRNAs in chicken embryo fibroblast (DF-1) cells under four conditions: control, MDV infection, chicken interferon-alpha (chIFN-α) treatment and MDV–chIFN-α co-treatment. Integrative bioinformatic analyzes were employed to identify key differentially expressed miRNAs (DEMs) and predict their target genes within the IFN-I signaling network.

**Results:**

MDV infection and chIFN-α treatment induced fundamentally distinct EV miRNA profiles. Strikingly, MDV infection counteracted the specific EV miRNA signature triggered by chIFN-α. We identified 65 key DEMs with the potential to cooperatively target multiple nodes of the IFN-I pathway. Among these, gga-miR-20a-5p and gga-miR-148a-3p were experimentally validated to directly target the 3′ untranslated regions of the key innate immune sensors cGAS and TLR3, respectively, leading to a significant suppression of downstream IFN-I signaling activation.

**Conclusion:**

This study identifies dysregulated EV miRNAs during MDV-interferon antagonism, validating their direct targeting of innate immune sensors. These findings provide new insights into viral pathogenesis and pinpoint specific miRNA-target axes as potential avenues for antiviral intervention.

## 1 Introduction

Members of the Herpesviridae family have evolved sophisticated mechanisms to evade host immune defenses, establishing persistent infections (Pei et al., 2020). Marek’s disease virus (MDV), an oncogenic alpha-herpesvirus, induces rapid-onset lymphomas in chickens (Nair, 2013; Hosseini et al., 2023). In 1907, the Hungarian veterinarian József Marek first identified the disease and described it as fowl paralysis, a generalized polyneuritis in chickens. His pioneering work laid the foundation for over a century of research on Marek’s disease virus (Honti and Lapis, 1977). The continuous emergence of hypervirulent strains under immune pressure poses a major threat to global poultry health (Zhang et al., 2022; Birhan et al., 2023), underscoring the need to decipher MDV’s immune evasion tactics.

Recent advances in intercellular communication research have revealed that virus-host interactions extend beyond intracellular compartments to include EVs-mediated pathways (Song et al., 2023; Wen et al., 2024). Small extracellular vesicles (sEVs), defined as EVs with diameters under 200 nm, serve as crucial mediators of intercellular communication by transporting bioactive cargo such as proteins, nucleic acids, and lipids that modulate recipient cell physiology (Conigliaro et al., 2017). Notably, sEVs are enriched with miRNAs, which can comprise 50% to 90% of total vesicular RNA (Agudelo et al., 2025). These vesicular miRNAs participate in immune regulation through targeted suppression of key signaling molecules. For instance, miR-146a negatively regulates the NF-κB pathway via TRAF6/IRAK1 inhibition (An et al., 2018), miR-21 modulates the STAT3-NF-κB signaling axis through PTEN targeting (Nguyen et al., 2021), and miR-302c directly suppresses NF-κB-inducing kinase (NIK) to influence innate immunity (Gui et al., 2015). Despite these advances, the potential involvement of sEV-derived miRNAs in MDV-host interactions remains largely unexplored.

Innate immunity serves as the first line of host defense against viral infections by inducing type I interferon (IFN-I) and a broad range of antiviral effectors (Ivashkiv and Donlin, 2014; Chen et al., 2017). Viral nucleic acids represent critical pathogen-associated molecular patterns that are recognized by host pattern recognition receptors (PRRs), thereby activating the IFN-I pathway. This cascade ultimately induces the expression of multiple interferon-stimulated genes (ISGs) and initiates innate antiviral responses (Qian et al., 2017; Muhammad et al., 2025). IFN-I response constitutes a cornerstone of antiviral innate immunity. To establish productive infection, virus has evolved multiple strategies to counteract IFN-I signaling. Recent studies have shown that MDV can evade the DNA-sensing pathway at various levels, including recognizing through multiple DNA sensors and innate immune signaling through the IRF7 or NF-κB axis (Li et al., 2019; Du et al., 2022). For example, viral proteins RLORF4, which inhibits NF-κB nuclear translocation to block cGAS-STING-mediated IFN-β production (Liu et al., 2019), virus protein kinase US3 inhibits DNA-sensing antiviral innate immunity via abrogating activation of NF-κB (Li et al., 2025). MDV inhibits IFN-β production by targeting DEAD/DEAH-box helicase 5 (DDX5) to interfere with the Toll-like receptor 3 signaling pathway, thereby promoting viral replication (Xu et al., 2021). However, existing research has predominantly focused on intracellular immune evasion mechanisms mediated by viral proteins, leaving open the question of whether MDV exploits sEVs as intercellular vehicles for immune modulation.

Here, we tested the hypothesis that MDV reprograms the sEV miRNA cargo to facilitate its escape from the IFN-I-mediated antiviral state. By comparative profiling of sEV miRNAs during MDV infection and chIFN-α challenge, we aimed to identify key miRNAs involved in this process. We further focused on candidates with the potential to target the IFN-I induction pathways, leading to the experimental validation of two sEV miRNAs that directly suppress the core sensors cGAS and TLR3. Our study uncovers a novel, EV-mediated dimension of herpesvirus immune evasion, providing fresh mechanistic insights and potential avenues for intervention.

## 2 Materials and methods

### 2.1 Cells and viruses

Primary chicken embryo fibroblast (CEF) cells were prepared from 9- to 11-day-old specific pathogen-free (SPF) chicken embryos (Hamburger-Hamilton stages 35-37). After decapitation and evisceration, the embryonic tissues were minced and digested with 0.25% trypsin-EDTA at 37 °C for 10–15 min with gentle agitation. The digestion was repeated 2–3 times, and the collected cell suspensions were filtered through a 70-μm cell strainer and centrifuged at 1,000 × g for 10 min at 4 °C. The cell pellet was resuspended in M199 medium supplemented with 10% fetal bovine serum (FBS), 100 U/mL penicillin, and 100 μg/mL streptomycin, and cultured at 37 °C in a humidified atmosphere with 5% CO_2_. These primary cells were used specifically for Marek’s disease virus (MDV) propagation and titration, as they support higher viral yields compared to cell lines.

DF-1 cells (Procell, CL-0279, China), an immortalized chicken fibroblast cell line, were cultured in Dulbecco’s Modified Eagle Medium (DMEM) containing 10% FBS, 100 U/mL penicillin, and 100 μg/mL streptomycin at 39 °C under 5% CO_2_. Due to their homogeneous nature and high transfection efficiency, DF-1 cells were employed for small extracellular vesicle (sEV) isolation, small RNA sequencing, and all subsequent functional validation experiments, including qPCR and dual-luciferase reporter assays.

### 2.2 MDV infection and IFN-α treatment experiments

The MDV-1 RB1B strain and chicken recombinant IFN-α used in this study were obtained from our laboratory repository (Arslan et al., 2019; Liu et al., 2020). The virus was propagated in CEF cells and tittered by plaque assay. Subsequent MDV infection and/or interferon-alpha (chIFN-α) treatment experiments were performed in DF-1 cells. Recombinant chicken chIFN-α was produced in-house using a baculovirus-silkworm expression system. The dosage and timing of interferon−α treatment, as well as the viral replication kinetics, were determined based on preliminary experiments. The dosage, timing, and viral replication kinetics of interferon−α treatment were determined based on preliminary experiments evaluating a range of concentrations (10–100 ng/mL) and time points (24–48 h), selecting conditions that induced stable antiviral responses without cytotoxicity.

Infection was performed at the 0.5 MOI for 2 h at 37 °C to allow viral adsorption. The inoculum was removed, the cells were washed twice with PBS, and fresh medium containing 2% FBS was added, as previously described (Boodhoo et al., 2019). Cells were incubated at 37 °C for 48 h. For IFN-I stimulation experiments, cells were treated with chicken recombinant IFN-α at a final concentration of 10,000 U/mL for 48 h. For the MDV and IFN-α co-treatment group, cells were first treated with 10,000 U/mL IFN-α for 24 h, followed by infection with 0.5 MOI MDV for an additional 24 h.

### 2.3 Preparation and characterization of sEVs

sEVs were isolated according to MISEV guidelines (Welsh et al., 2024). Briefly, cell culture supernatants were sequentially centrifuged (300 × g, 10 min; 2,000 × g, 15 min; 10,000 × g, 30 min) to remove debris and concentrated using a 100 kDa ultrafiltration device. The concentrate was ultracentrifuged (100,000 × g, 70 min; Ti45 rotor), and the pellet was resuspended in PBS, filtered through a 0.22 μm membrane, and subjected to a second ultracentrifugation under the same conditions. The final sEV pellet was resuspended in PBS and characterized using a Videodrop particle analyzer for concentration and size distribution (Llorens-Revull et al., 2023).

### 2.4 Transmission electron microscopy

TEM (Hitachi, Japan) was used to characterize the morphology of isolated sEVs. Purified sEVs (1 μg) were adsorbed onto a carbon-coated copper grid for 2 min, washed with distilled water, and negatively stained with 2% uranyl acetate. Samples were imaged using a TEM operating at 120 kV.

### 2.5 Nanoparticle tracking analysis

The size and concentration Characterization of sEVs were assessed using the Corning^®^ Videodrop system (Moccia et al., 2023). Briefly, 10 μL of sEVs resuspended in PBS was placed on a specialized Videodrop slide within a magnetic sample holder. Real-time analysis of nanoparticle concentration and Brownian motion was initiated by selecting “record.” Three independent measurements were performed for each sample.

### 2.6 Western blot analysis of sEVs and whole-cell lysates

sEVs were lysed on ice for 10 min using an equal volume of lysis buffer, followed by centrifugation at 12,000 rpm for 10 min at 4 °C. The supernatant was mixed with SDS loading buffer, denatured at 95 °C for 10 min, and separated by SDS-PAGE (10% gel). After transfer to a polyvinylidene fluoride (PVDF) membrane (Merck Millipore, Germany), blots were probed overnight at 4 °C with primary antibodies against TSG101, RAB1A, and Hsp90, followed by incubation with appropriate secondary antibodies (Dangot et al., 2023). Protein signals were visualized by chemiluminescence using a chemiluminescence imager (e-Blot, China) with ECL reagents (Yeason, China).

### 2.7 Small RNA library construction, sequencing, and miRNA data processing

The experimental procedures were performed following the standard Illumina protocol, which included library preparation and sequencing experiments. Small RNA sequencing libraries were prepared using the TruSeq Small RNA Sample Prep Kits (Illumina, San Diego, USA). Upon completion of library construction, the resulting libraries were sequenced on an Illumina HiSeq 2000/2500 platform to generate single-end 50-bp reads.

miRNA data analysis was performed using the ACGT101-miR (v4.2) pipeline (LC-Bio, China) (Han X. et al., 2016; Jeon et al., 2019). The analytical workflow consisted of the following key steps (Pei et al., 2020): Raw reads were processed to remove 3’ adapters and low-quality sequences, yielding clean data (Hosseini et al., 2023); small RNA length filtering was applied, retaining sequences of 18–26 nt (animals) (Nair, 2013); the remaining sequences were aligned against mRNA, Rfam, and Repbase databases (excluding miRNA databases) for annotation and filtering; and (Honti and Lapis, 1977) miRNA identification was conducted by mapping the filtered reads to precursor sequences and reference genomes to annotate known miRNAs and predict novel candidates. Raw sequencing data were processed through base calling to generate FASTQ files.

Small RNA sequencing was performed on three independent biological replicates per condition (n = 3). Differentially expressed miRNAs were identified using DESeq2 with a threshold of |log2(fold change)| > 1 and adjusted P value < 0.05. Multiple testing correction was applied using the Benjamini-Hochberg method to control the false discovery rate (FDR).

### 2.8 miRNA identification and expression analysis

miRNA identification was performed by aligning length-filtered reads to the miRBase database and the chicken reference genome using the ACGT101-miR pipeline. miRNA expression levels were quantified and normalized to generate normalized expression values, designated as norm values (Zhang et al., 2020), which served as the primary metric for assessing miRNA abundance across different samples. Statistical analysis was conducted on these normalized expression values to assess intra- and inter-group correlations and identify DEMs.

### 2.9 Screening of DEMs

DEMs were identified based on fold change and statistical significance (Li et al., 2016). Analysis was performed using normalized expression values. A p-value computation model based on the normal distribution was applied, with significance set at p < 0.05. Student’s t-test was used for two-group comparisons, and one-way ANOVA was used for multiple-group comparisons (Wang et al., 2020; Han et al., 2022).

### 2.10 Prediction and function enrichment analysis of miRNA targets

Gene targets of candidate miRNAs were predicted using TargetScan (v5.0) and miRanda (v3.3a) (Agarwal et al., 2015; Hu et al., 2021). Targets were filtered by context score percentile ≥ 50 for TargetScan and maximum free energy < −10 kcal/mol for miRanda. Only overlapping targets were retained. GO and KEGG enrichment analyzes were performed using DAVID (Chen and Wang, 2020), with significance determined by hypergeometric test and Benjamini–Hochberg correction (Q-value < 0.05).

### 2.11 Real-time RT-PCR analyzes

qRT-PCR was performed according to established methods (Wang et al., 2023). Briefly, cDNA was synthesized from miRNA using a stem-loop cDNA Synthesis Kit (Vazyme, China) and amplified using Universal SYBR Green Supermix (Vazyme, China) on a 7500 Real-Time PCR System (Thermo Fisher, USA). Primers are listed in **Supplementary Table 8**. All reactions were run in triplicate using validated primers (**Supplementary Table 8**). miRNA expression was normalized to U6 snRNA and analyzed using the 2^−ΔΔCT^ method (Kramer, 2011).

### 2.12 Dual-luciferase reporter assay

A partial 3′UTR (~400 nt) of the target gene containing predicted miRNA-binding sites was cloned into the pmiR-report vector (Kaur et al., 2022). A mutant construct with altered binding sequences served as the control (Stroynowska-Czerwinska et al., 2014). Plasmids, miRNA mimics, and negative controls were synthesized by Rui Biotech (Beijing, China). DF-1 cells were seeded in 24-well plates and co-transfected at 75%–85% confluence with 50 nM miRNA mimic (or negative control) and 1.0 µg plasmid using Lipofectamine 2000 (Thermo Fisher, USA). After 48 h, luciferase activity was measured using the Dual-Luciferase Reporter Assay System (Promega, USA), and firefly luciferase activity was normalized to Renilla (Zhu et al., 2023).

### 2.13 Statistical analysis and visualization

Graphs and statistical analyzes were performed using GraphPad Prism software (version 9.5; La Jolla, CA, USA). Data derived from at least three independent experiments with three replicates each are presented as the mean ± SD. Significant differences in gene expression and luciferase activity between the treated and control groups were determined and analyzed using the analysis of variance (ANOVA).

## 3 Results

### 3.1 Isolation and characterization of EVs from conditioned cell culture medium

**Figure 1:**
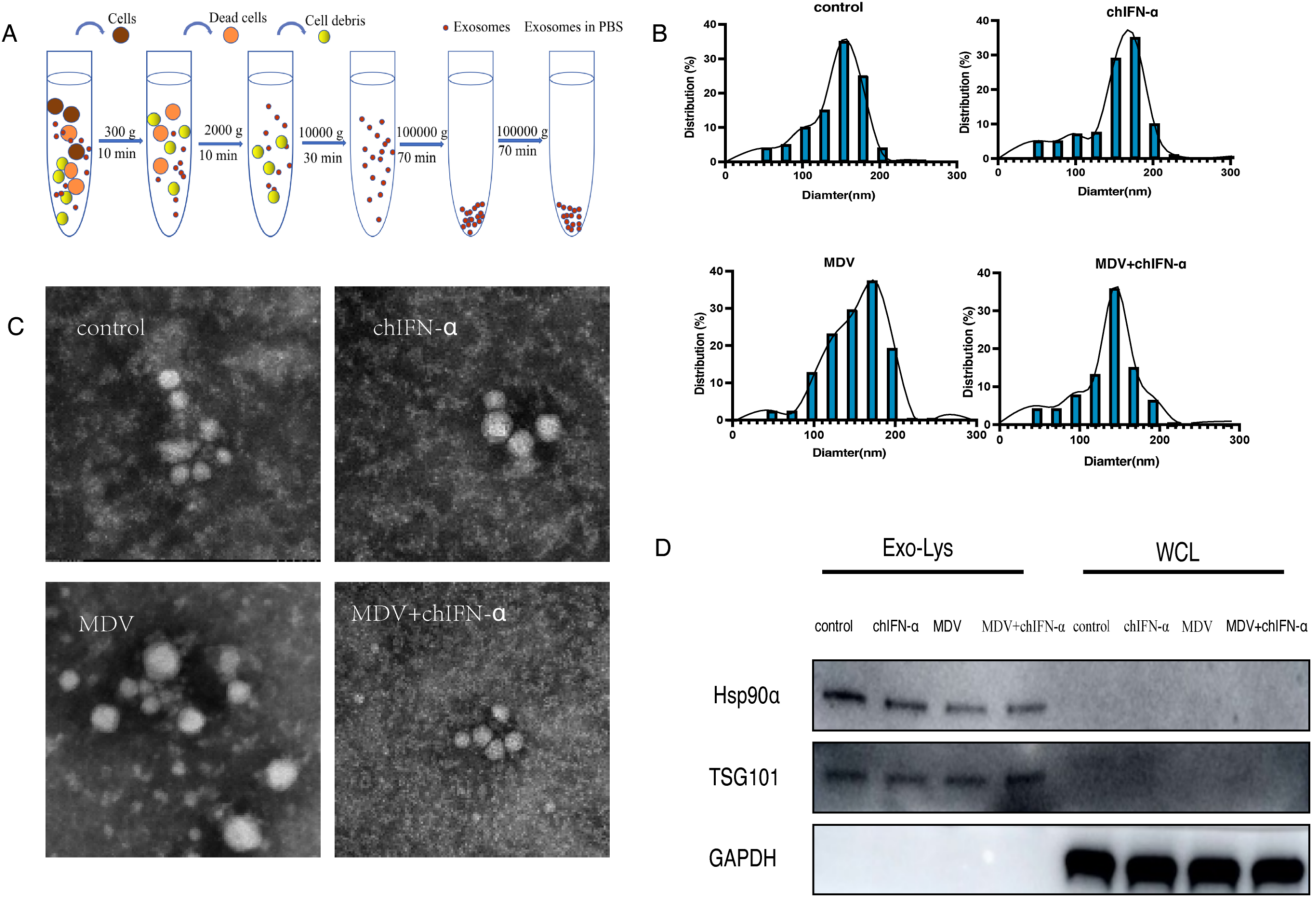
Characterization of EVs purified from DF-1 cell culture supernatant. **(A)** Schematic diagram of the isolation and purification procedure for EVs from the DF-1 cell culture supernatant. **(B)** Nanoparticle tracking analysis showing the size distribution of the isolated EVs, with most particles falling within the typical size range for vesicles. **(C)** Transmission electron micrograph showing that the purified vesicles exhibit the classic cup-shaped or spherical morphology with an intact membrane structure. **(D)** Western blot analysis confirming the presence of the EV marker proteins TSG101 and Hsp90α in the preparations.

EVs were isolated from the culture supernatants of DF-1 cells representing all experimental groups, including control, MDV-infected, chIFN-α-treated, and MDV plus chIFN-α co-treated groups, using a multi-step protocol combining ultrafiltration and differential centrifugation with forces ranging from 300 × g to 100,000 × g (**Figure 1A**). The purified EVs underwent comprehensive characterization through TEM, nanoparticle tracking analysis, and Western blotting. Nanoparticle tracking analysis and TEM of the isolated sEVs revealed a size distribution of 30–200 nm and the absence of contaminating cellular debris or viruses (**Figures 1B, C**). Furthermore, Western blot analysis showed strong enrichment of the EV-specific markers Hsp90α and TSG101 in EV fractions compared with whole-cell lysates, confirming the high purity and characteristic properties of the isolates (**Figure 1D**). Together, these results demonstrate the successful isolation of high-purity extracellular vesicles (EVs) suitable for subsequent small RNA analysis.

### 3.2 Correlation and differential analyzes of miRNA expression

**Figure 2:**
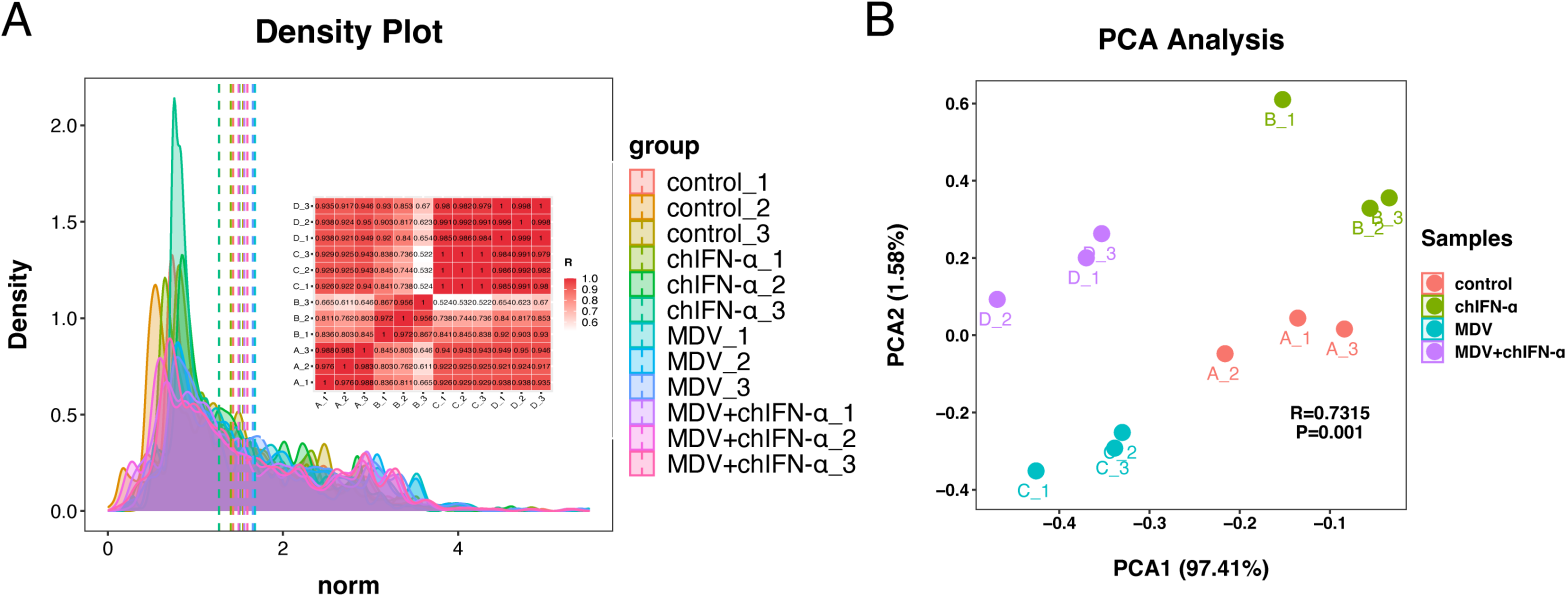
Correlation analysis of miRNA expression levels across samples. miRNA expression levels in each sample were normalized and subjected to statistical analysis. **(A)** TPM density distribution patterns (shaded line plots) and Pearson correlation coefficients between samples are shown. A correlation coefficient (R) closer to 1 indicates higher similarity between samples within a treatment group. The ENCODE Consortium recommends that, under ideal experimental conditions, the squared Pearson correlation coefficient should exceed 0.92. The intrinsic heterogeneity of extracellular vesicle miRNAs may result in lower intra-group correlations compared with inter-group correlations. **(B)** Principal component analysis (PCA) plot. The horizontal (PCA1) and vertical (PCA2) axes represent the first and second principal components with the highest contributions, respectively. The percentage of explained variance for each component is indicated on the axis labels. PCA1 accounts for 97.33% of the total variance, indicating that data variation is highly concentrated along a single dimension. PCA2 contributes only 1.93%, suggesting minimal influence from secondary factors. The proximity between points in the plot reflects similarity in sample composition. The close clustering of samples within the same group and their distinct separation from other groups indicate strong biological reproducibility and homogeneity among replicates.

Correlation and principal component analysis (PCA) of miRNA expression revealed clear sample clustering and high experimental reproducibility (**Figure 2**). The density distribution plot showed broadly overlapping profiles across all 12 samples, indicating that EVs from each treatment group maintained basal expression levels for most miRNAs. miRNAs in the high-expression region displayed greater dispersion, consistent with expression changes triggered by MDV infection and chIFN-α treatment. Pearson correlation analysis further demonstrated high intra-group reproducibility, with correlation coefficients exceeding 0.92 for all biological replicates except between chIFN-α-3 and chIFN-α-2 (r=0.867). PCA confirmed that all samples, including chIFN-α-3, clustered appropriately within their respective groups (**Figure 2B**), supporting its inclusion in subsequent analyzes. Notably, the three treatment groups were clearly separated from the control group along the first principal component (PCA1), highlighting distinct, group-specific miRNA expression patterns.

### 3.3 Comparable total and unique small RNA compositions across treatment groups

**Figure 3:**
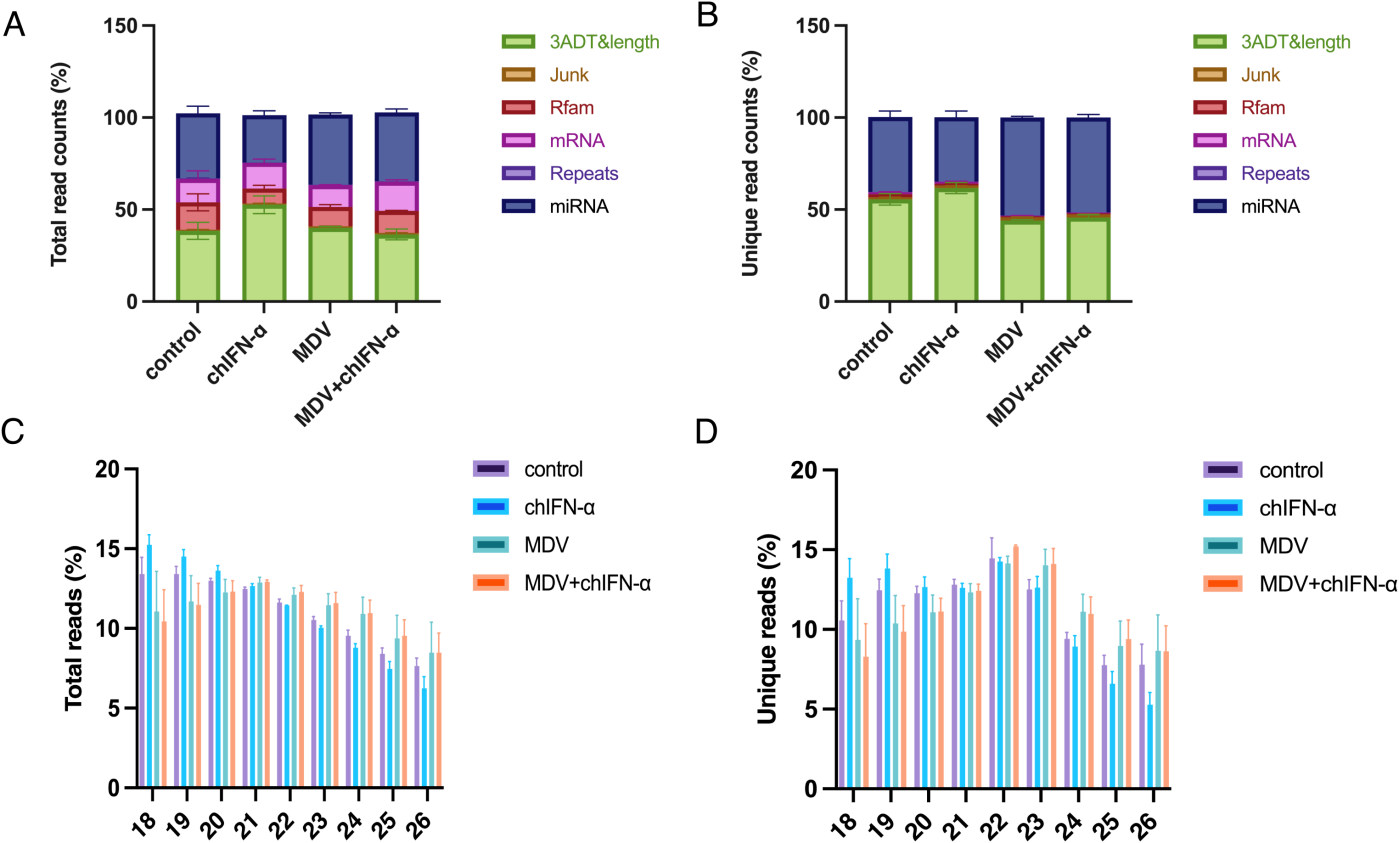
Classification and composition profiles of sRNAs in extracellular vesicle samples obtained by sRNA-Seq. **(A)** Annotation of total sequence counts for small RNAs in extracellular vesicles across different treatment groups. **(B)** Annotation of unique small RNA species in extracellular vesicles across different treatment groups. **(C)** Length distribution of total miRNA sequences in extracellular vesicles across different treatment groups. **(D)** Length distribution of unique miRNA species in extracellular vesicles across different treatment groups.

**Figures 3A, B** summarize the key characteristics of the quality-controlled sRNA-seq data and the composition of small RNAs across the experimental groups. All samples yielded over 20 million high-quality reads, providing sufficient depth for comprehensive sRNA profiling and robust cross-group comparisons. Annotation results indicated that approximately 50% of unique reads in sEV samples were mapped to miRNAs (**Figure 3A**; **Supplementary Figure S1**). Comparative analysis further revealed that the highest proportion of miRNAs relative to total sRNAs occurred in the MDV-infected and co-treatment groups, whereas the chIFN-α-treated group exhibited the lowest miRNA representation. As shown in **Figures 3C, D**, the length distribution of sequenced miRNAs (18–26 nucleotides) displayed consistent and reproducible patterns across all samples, following stringent filtering and normalization of sequence read counts and distinct miRNA species.

### 3.4 Known and novel miRNA profiled in each treatment groups

**Figure 4:**
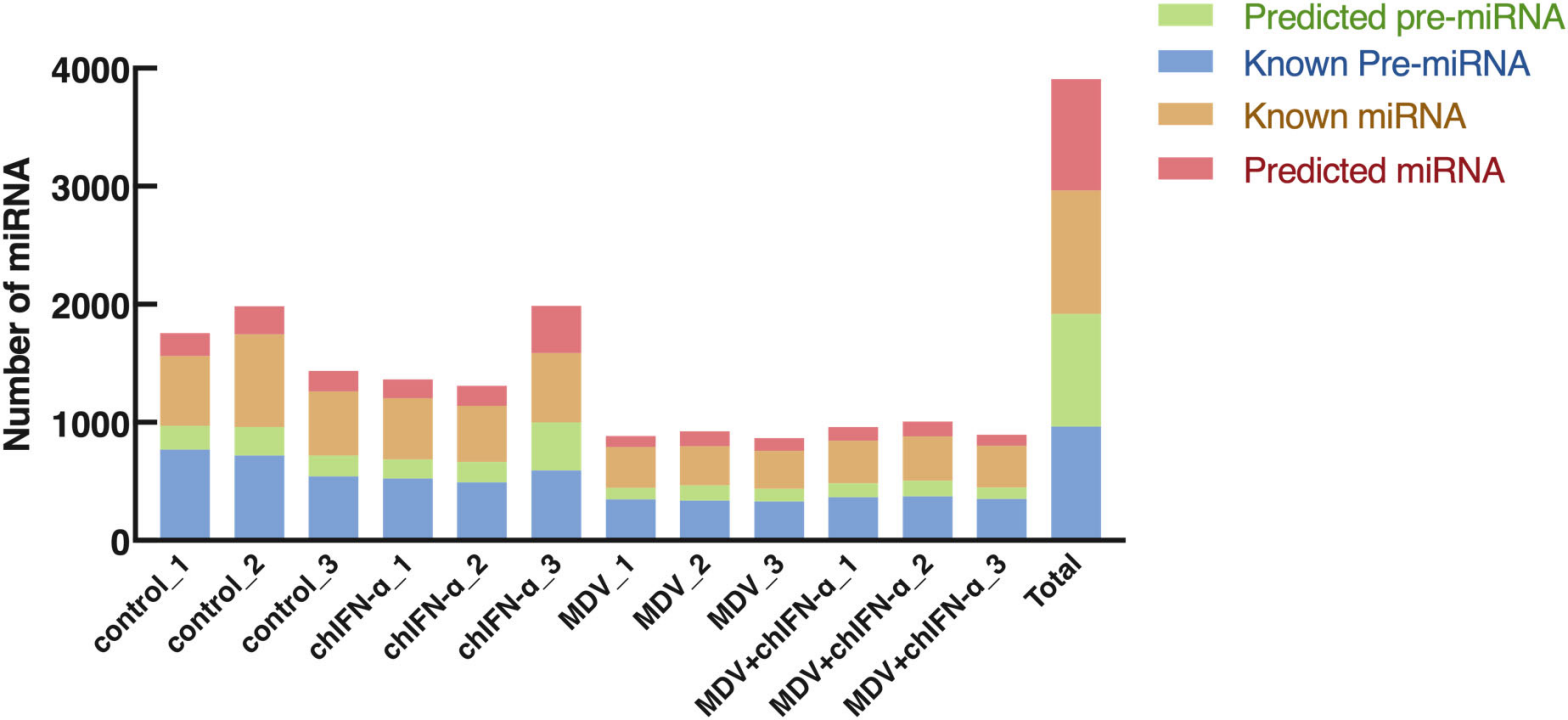
Profiling of known and novel miRNAs in sEVs. The chart illustrates the abundance of known miRNAs (aligned to annotated precursors in miRBase: https://www.mirbase.org/) and novel predicted miRNAs. Novel miRNAs were identified as reads that did not map to any known pre-miRNAs in miRBase but could be aligned to the host reference genome.

As summarized in **Figure 4**, high-throughput sequencing of sEV samples yielded over one million small RNA reads, with approximately 90% mapping successfully to the 1,043 known miRNA stem-loop precursors and 1,281 corresponding mature miRNA sequences annotated in the chicken genome. While most miRNAs were detected at low abundance—ranging from one to several copies—a subset of highly expressed miRNAs exhibited read counts exceeding 10,000 (**Supplementary Table 1**). In addition, MDV-encoded viral miRNAs were also detected in the sequencing results, totaling 10 species (**Supplementary Table 2**). Although this study focuses primarily on host miRNAs, the potential role of these viral miRNAs in immune evasion warrants further investigation. Together, these findings confirm that sRNA-seq robustly captures a broad spectrum of small RNA species in sEVs, from rare transcripts to highly abundant miRNAs, including those that are ubiquitously or differentially expressed under varying conditions.

### 3.5 Validation of sRNA-seq data by quantitative real-time PCR

**Figure 5:**
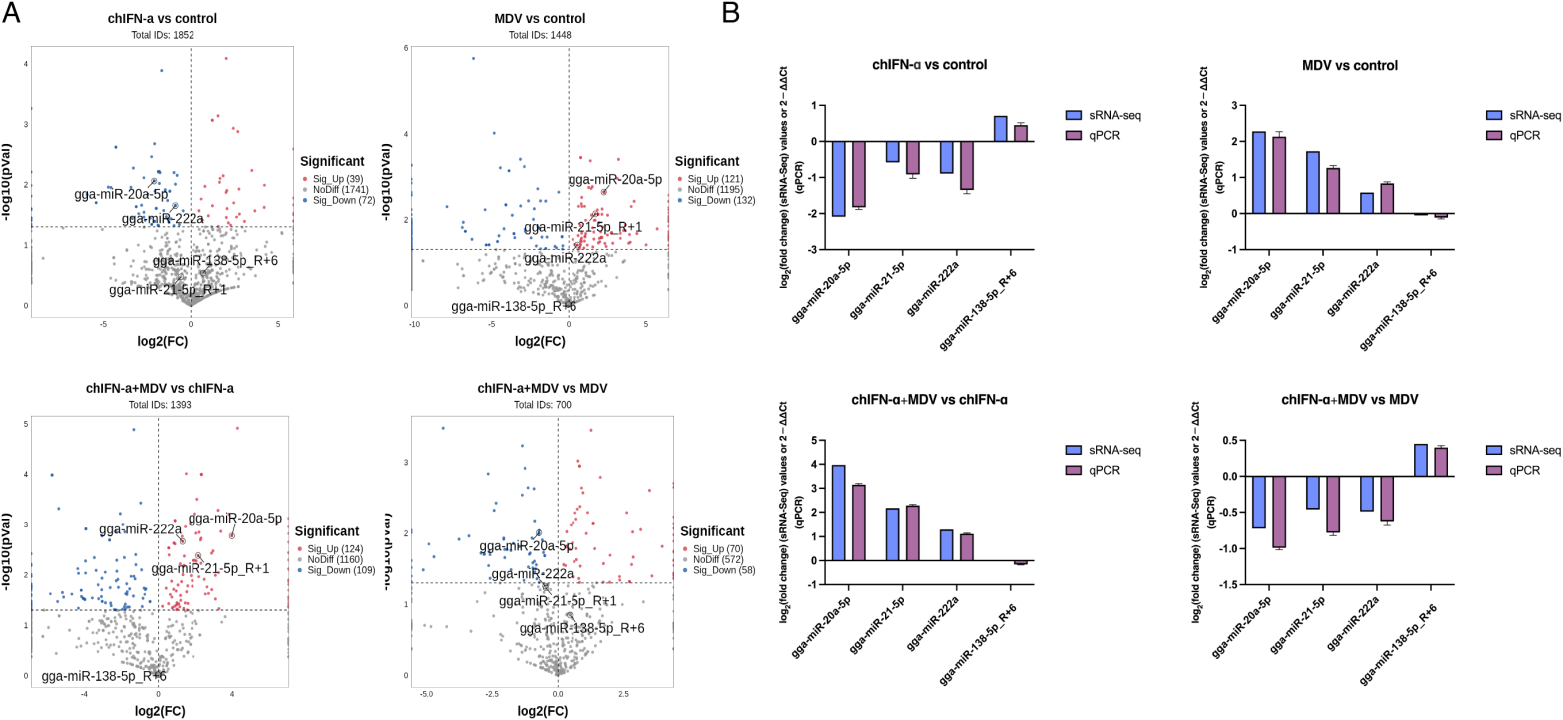
Validation of the reliability of high-throughput sequencing data. **(A)** Volcano plots displaying pairwise comparisons between the following groups: control vs. MDV-infected, control vs. chIFN-α-treated, co-treated (MDV + chIFN-α) vs. MDV-infected, and co-treated vs. chIFN-α-treated. Significantly upregulated miRNAs (p < 0.05) are shown in red, downregulated miRNAs in blue, and non-significant miRNAs in gray. **(B)** Differential expression of selected known and novel miRNAs identified by sRNA-Seq was verified using target-specific qPCR. The y-axis represents the relative expression level expressed as 2^−ΔΔCt^ for qPCR data or log_2_(fold change) for sequencing data. The 2^−ΔCt^ and 2^−ΔΔCt^ values were calculated by normalizing target gene Ct values to the Ct values of U6 small nuclear RNA from the same RNA sample. The data demonstrate overall consistency between the sRNA-Seq and qPCR results.

To validate the reliability of our sequencing data, we performed quantitative real-time PCR (qPCR) analysis on four randomly selected miRNAs in sEVs from the control and treatment groups. To account for EV heterogeneity, we selected four random miRNAs namely, gga-miR-20a-5p, gga-miR-21-5p_R+1, gga-miR-222a, and gga-miR-138-5p_R+6, whose expression levels and statistical significance in the sRNA-seq dataset included both highly and lowly expressed species (**Figure 5A**; **Supplementary Tables 3**-**6**). The qPCR results confirmed the robustness of the sRNA-seq data, as summarized in **Figure 5B**. Specifically, gga-miR-20a-5p, gga-miR-21-5p_R+1, and gga-miR-222a displayed significant upregulation after MDV infection, significant downregulation following chIFN-α treatment, and marked upregulation under co-treatment conditions, all relative to the control. Expression trends of gga-miR-138-5p_R+6 measured by qPCR were also in overall agreement with the sRNA-seq profiles, further supporting the consistency between the two platforms.

### 3.6 MDV infection and chIFN-α treatment induce opposing sEV miRNA profiles

**Figure 6:**
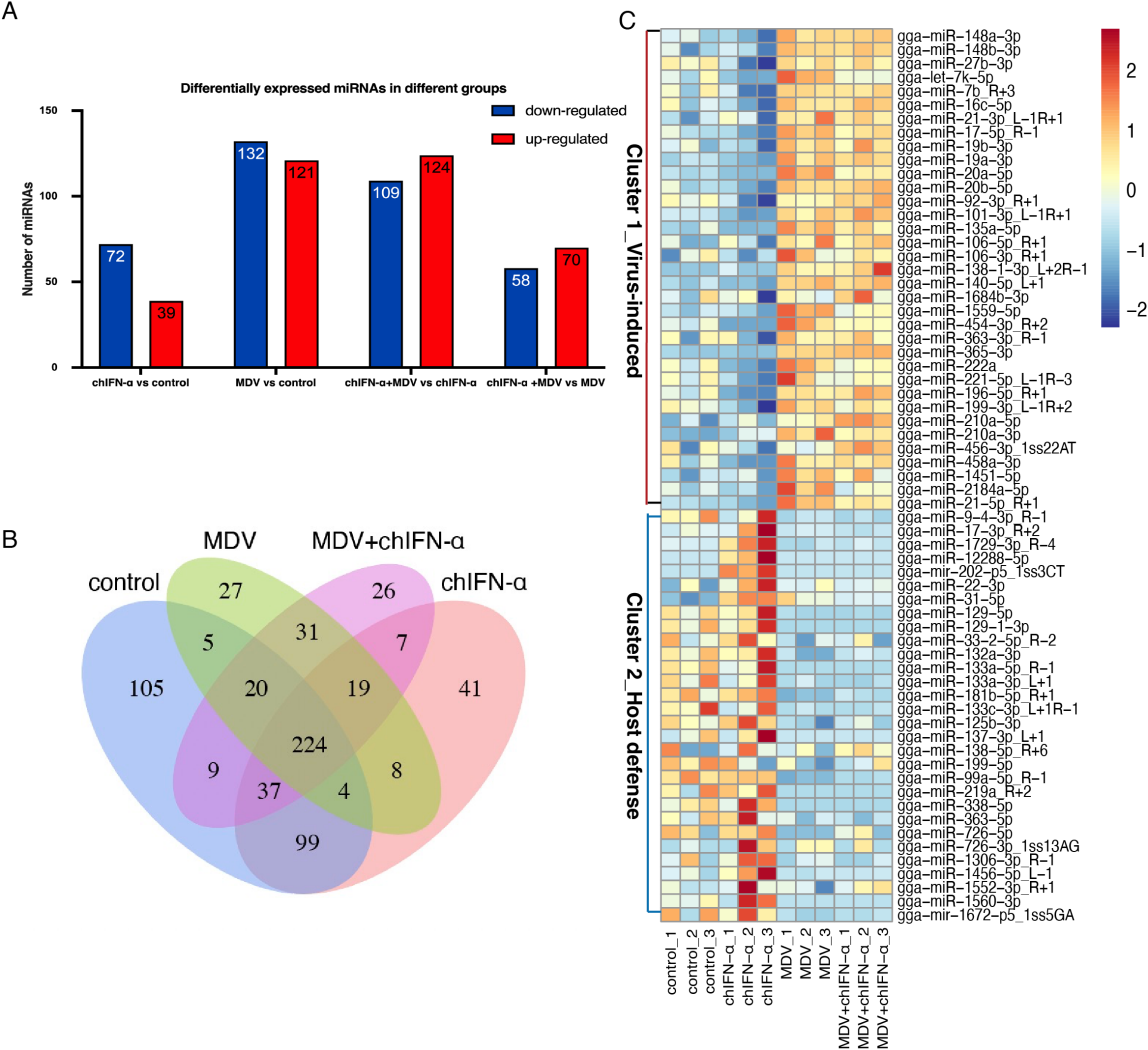
Differential expression and cluster analysis of miRNAs across experimental groups. **(A)** Grouped bar chart illustrating the numbers of differentially expressed miRNAs across the four experimental comparisons. The number of differentially expressed miRNAs with P value < 0.05 was counted. The x−axis indicates the comparison groups, and the y−axis indicates the number of up−regulated and down−regulated miRNAs. Red bars represent up−regulated miRNAs, and blue bars represent down−regulated miRNAs. The numbers on the bars indicate the respective counts of up−regulated and down−regulated miRNAs. **(B)** Venn diagram illustrating the overlap of DEMs among the different treatment groups. **(C)** Heatmap depicting expression patterns of 65 DEMs (p < 0.05) across samples. miRNAs are grouped by hierarchical clustering based on similar expression profiles. Color intensity represents normalized expression levels, with red/orange indicating high expression and blue indicating low expression. Heatmaps were generated using the OmicStudio platform (https://www.omicstudio.cn).

MDV infection and chIFN-α induce opposing miRNA profiles, with MDV counteracting the interferon signature. **Figure 6A** presents a grouped bar chart showing the numbers of upregulated and downregulated differentially expressed miRNAs among the four experimental groups. As shown, in the MDV infection group, 121 miRNAs were upregulated and 132 were downregulated. In the IFN-α treatment group, 39 miRNAs were upregulated and 72 were downregulated. In the MDV+IFN-α co-treatment group, compared with the IFN-α treatment group, 124 miRNAs were upregulated and 109 were downregulated; compared with the MDV infection group, 70 miRNAs were upregulated and 58 were downregulated. The Venn diagram in **Figure 6B** clearly shows the overlap of miRNAs under different conditions, and **Supplementary Table 9** summarizes their up−regulation and down−regulation in these conditions. This intuitively compares the direction of miRNA expression changes under different treatment conditions, providing a basis for subsequent screening of candidate miRNAs with specific regulatory trends.

To identify miRNAs central to this virus-host interplay, we focused on 65 DEMs that were consistently detected across all conditions but exhibited marked variation. A subset of these, showing perfectly antagonistic responses to MDV and chIFN-α, clearly segregated into two clusters: Cluster 1 (Virus-induced): miRNAs with low baseline expression were strongly suppressed by chIFN-α but markedly upregulated by MDV infection, even in the presence of chIFN-α during co-treatment; Cluster 2 (Host-defense): miRNAs with high basal expression were further enhanced by chIFN-α but significantly suppressed by MDV infection, regardless of co-treatment (**Figure 6C**; **Supplementary Figure S3**). This precise antagonism identifies Cluster 1 miRNAs as prime candidates for viral subversion of the host type I interferon response, as the target genes of Cluster 1 miRNAs are mainly enriched in the MAPK signaling pathway.

### 3.7 Functional enrichment implicates a coordinated miRNA network targeting immune and cellular homeostasis pathways

**Figure 7:**
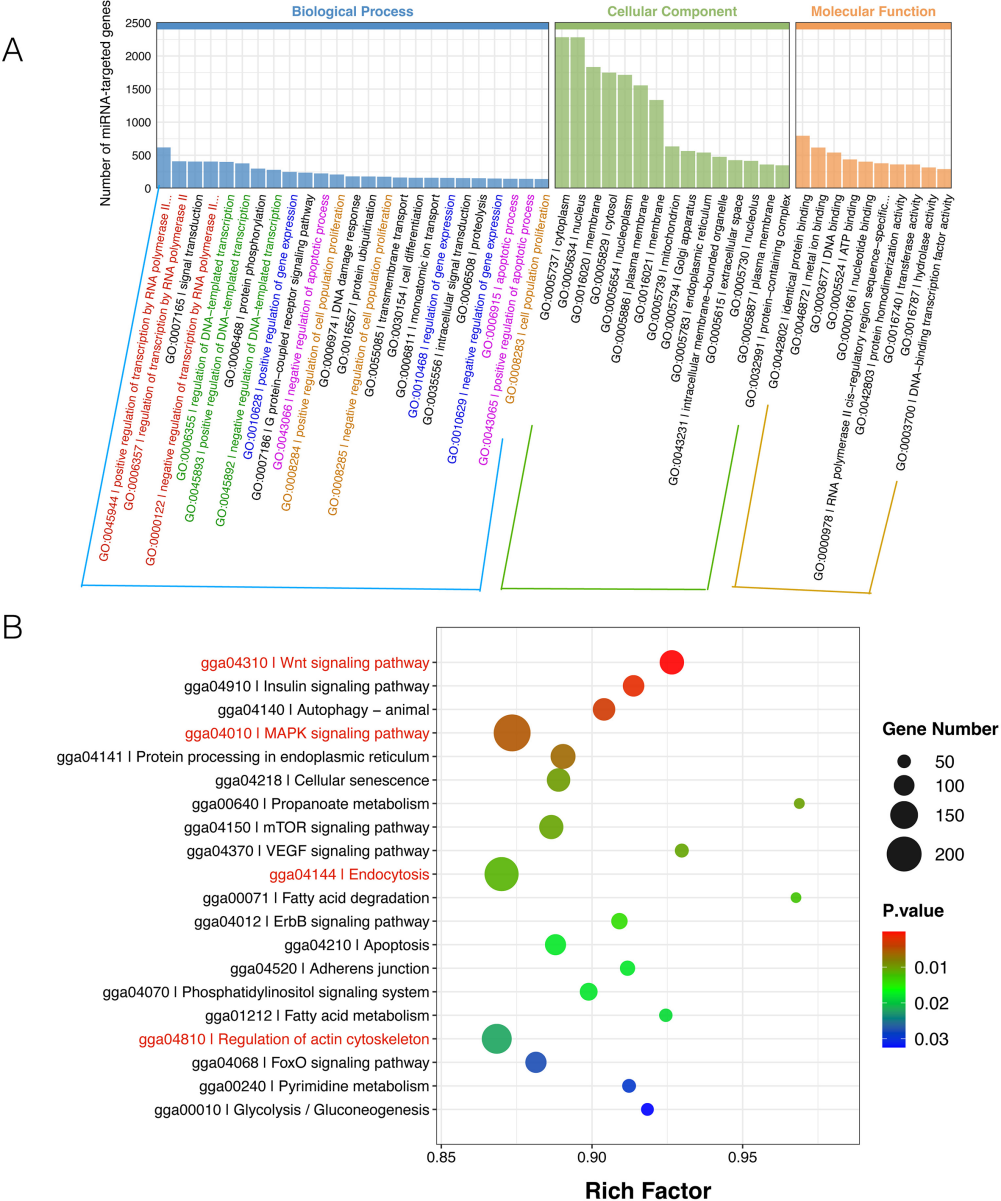
Functional enrichment analysis of predicted target genes for differentially expressed miRNAs. **(A)** GO analysis of miRNA target genes across three main categories: cellular component (CC), molecular function (MF), and biological process (BP). Terms shown meet the significance threshold of adjusted p < 0.05. **(B)** Pathway enrichment analysis of target genes for differentially expressed miRNAs. The y-axis lists significantly enriched pathways, with bubble size representing the number of target genes associated with each pathway.

Gene Ontology (GO) enrichment analysis was performed using all differentially expressed miRNAs identified from all experimental comparisons. The analysis revealed that the predicted targets of these DEMs were significantly enriched across biological processes, molecular functions, and cellular components (**Figure 7A**). Within biological processes, target genes were heavily involved in central signaling and regulatory functions, including intracellular signal transduction, transcriptional regulation, apoptosis, and cell proliferation. Notably, for core processes such as transcription and apoptosis, target genes were almost equally distributed between positive and negative regulators. This pattern suggests that MDV may orchestrate a precise recalibration of host cell state by simultaneously deploying miRNAs that suppress proviral pathways while repressing miRNAs that otherwise enhance antiviral defenses. In addition, GO enrichment analysis performed separately on the DEMs of each experimental comparison yielded similar results, as shown in **Supplementary Figure 4**.

KEGG pathway analysis, also based on the same complete set of DEMs, revealed that the predicted target genes of the differentially expressed miRNAs were significantly enriched in several key signaling pathways (**Figure 7B**). These included the MAPK signaling pathway and the Wnt signaling pathway, both of which are established regulators of the type I interferon response. Furthermore, the endocytosis pathway, which is essential for the biogenesis and cellular uptake of extracellular vesicles, was also among the most significantly enriched. This pattern of enrichment indicates that the MDV-altered EV miRNAs possess the collective potential to regulate not only core immune signaling pathways but also the fundamental processes governing EV-mediated intercellular communication. Moreover, KEGG pathway enrichment analysis performed separately on the DEMs of each experimental comparison revealed that miRNAs differentially expressed in response to MDV infection were largely involved in cellular metabolism, whereas those differentially expressed following chIFN−α treatment were mostly enriched in the MAPK pathway. Enrichment analyzes of the co−treatment group versus either the MDV infection group or the chIFN−α treatment group further supported these observations (**Supplementary Figure 5**).

### 3.8 MDV-upregulated sEV miRNAs directly target and suppress key sensors of the IFN-I pathway

**Figure 8:**
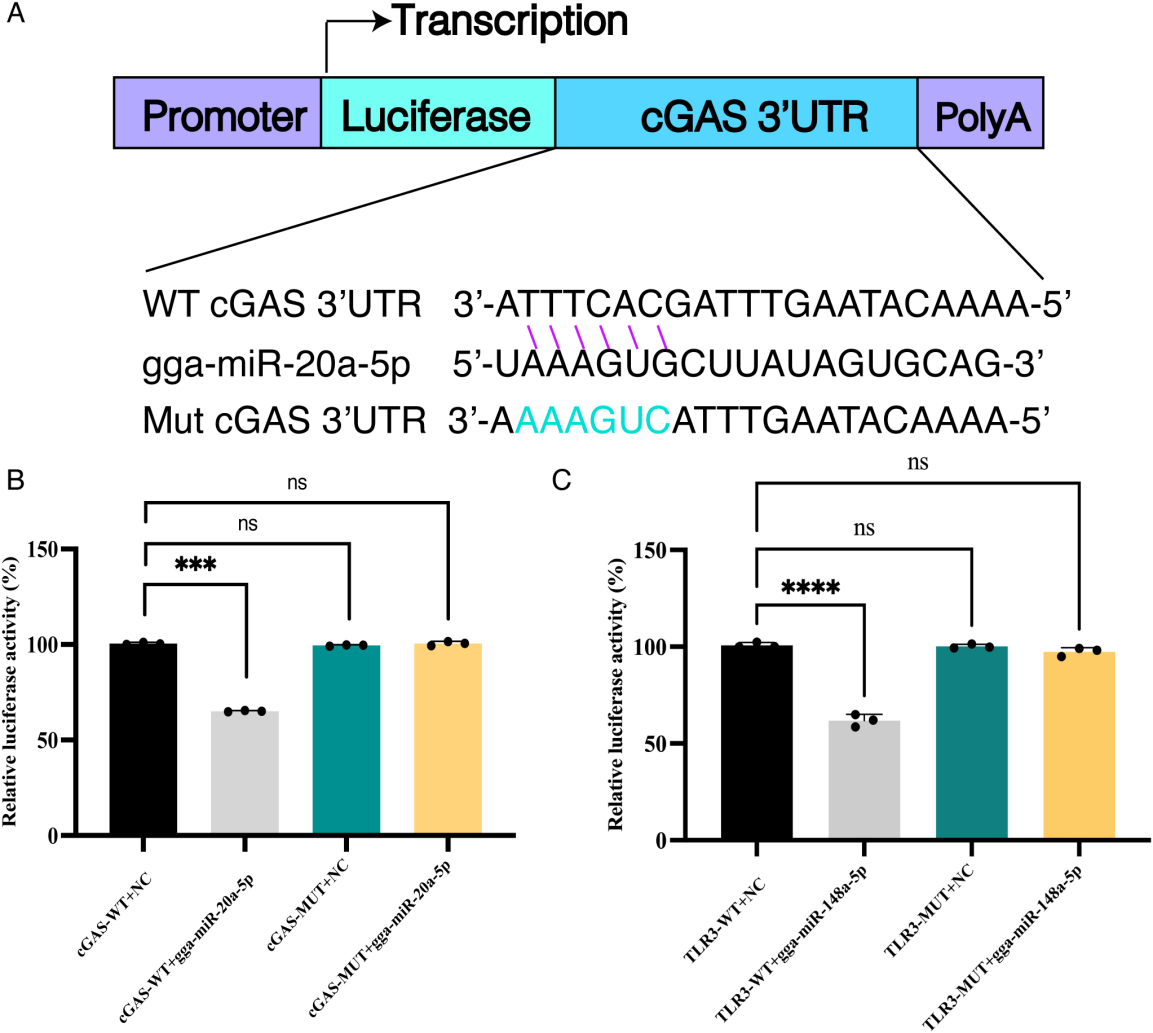
Validation of miRNA–target interactions using a dual-luciferase reporter assay. **(A)** Schematic diagram of the assay procedure, illustrated using gga-miR-20a-5p and cGAS as an example. Targeting relationships were verified using synthetic siRNA mimics with sequences fully complementary to the selected miRNAs or specific miRNA inhibitors. **(B, C)** Statistical significance is indicated as “***” for p < 0.01 and “****” for p < 0.005 (n = 3), representing differences relative to mimic-transfected control cells.

To test whether the MDV-induced sEV miRNAs functionally impair innate immunity, we focused on candidates predicted to target the 3′UTRs of core IFN-I signaling components (**Table 1**; **Supplementary Table S7**), including the cytosolic DNA sensor cGAS and the RNA sensor TLR3. DF-1 cells were selected for the dual-luciferase reporter assay on the basis of their ease of culture and transfection. Dual-luciferase reporter assays confirmed that gga-miR-20a-5p and gga-miR-148a-3p directly bind to the 3′UTRs of cGAS and TLR3, respectively, leading to significant suppression of reporter activity (**Figure 8**, p < 0.01). Critically, both miRNAs are members of Cluster 1—the subset of miRNAs that are specifically suppressed by chIFN-α but strongly and selectively upregulated during MDV infection (**Figure 5C**). This cluster-specific origin provides direct evidence that the viral manipulation of the sEV miRNA cargo is non-random and functionally coherent. Together, these results demonstrate that MDV achieves immune evasion, at least in part, through the coordinated upregulation of specific sEV miRNAs that silence the pivotal upstream sensors of the host IFN-I system.

**Table 1:** miRNAs with shared seed sequences targeting key genes in the IFN-I signaling pathway.

miR_Name^A^	Sequence (5’-3’)^B^	Target Gene^C^	Score^D^
gga-miR-20a-5p	U**AAAGUGC**UUAUAGUGCAGGUA	cGAS/JAK1/IFNAR1	99/85/68
gga-miR-17-5p	C**AAAGUGC**UUACAGUGCAGGUAGU	cGAS/JAK1/IFNAR1	99/91/68
gga-miR-106-5p	A**AAAGUGC**UUACAGUGCAGGUA	cGAS/JAK1/IFNAR1	99/88/68
gga-miR-19a-3p	U**GUGCAAA**UCUAUGCAAAACUGA	IFNAR1	99
gga-miR-148a-3p	A**AAGUUCU**GUGACACUCAGACU	TLR3	93

(A) Name of the miRNA targeting key components of the type I interferon signaling pathway. (B) Sequence of the mature miRNA (5’ to 3’), with the seed sequence bolded. (C) Name of the target gene within the type I interferon signaling pathway. (D) Targeting score predicting the miRNA-gene interaction.

## 4 Discussion

The evolutionary arms race between viruses and host immunity has driven the development of sophisticated immune evasion strategies across the viral kingdom (Chen J. et al., 2024; Chen N. et al., 2024). Among these, the manipulation of host miRNAs has emerged as a conserved mechanism, with viruses either encoding their own miRNAs to suppress host defenses or hijacking host miRNAs to create a favorable replication environment (Ringel et al., 2019; Van Royen et al., 2022). However, most studies to date have focused on cell-autonomous mechanisms, wherein viral proteins or miRNAs function within the infected cell itself (Ostrycharz and Hukowska-Szematowicz, 2022; Powell et al., 2025). The discovery that EVs can serve as carriers of regulatory miRNAs has introduced an entirely new dimension to this interplay: the potential for viruses to exert immune-modulatory effects at a distance, preemptively conditioning recipient cells before they encounter the virus directly.

Recent studies have shown that viral infection can alter the composition of host factors in extracellular vesicles, and that virus-encoded miRNAs can be selectively loaded into EVs, thereby influencing bystander cells without direct infection. For example, HSV-1-encoded miR-H28 and miR-H29 are secreted via exosomes and induce IFN-γ production in uninfected cells (Han Z. et al., 2016), suggesting that EV-mediated transfer of viral miRNAs may represent a general mechanism by which herpesviruses modulate host immunity. However, whether Marek’s disease virus, a strictly cell-associated avian alphaherpesvirus, also utilizes this pathway remains unclear. To systematically evaluate the impact of MDV infection on the sEV miRNA profile, we performed small RNA sequencing analysis. The results showed that MDV infection significantly altered the expression of 253 miRNAs, a number more than double that induced by chIFN-α treatment alone. More importantly, the miRNA expression profiles induced by MDV and chIFN-α treatment exhibited a broad antagonistic relationship: in the co-treatment group, MDV reversed most of the miRNA changes induced by chIFN-α alone. These observations indicate that MDV reprogramming of the sEV miRNA cargo is not a passive consequence of cellular stress or viral replication, but rather an active immune evasion strategy. This is consistent with emerging evidence that other viruses also manipulate EV cargo to facilitate immune evasion. For example, human cytomegalovirus (HCMV) has been shown to package specific miRNAs into EVs that target immune-related genes in recipient cells, thereby modulating the host antiviral response (Streck et al., 2020; Martin et al., 2026). Similarly, Epstein-Barr virus (EBV)-encoded miRNAs are selectively incorporated into EVs and can be transferred to non-infected cells, where they suppress immune recognition pathways (Cone et al., 2019; Hu et al., 2025). More recently, hepatitis C virus (HCV) was reported to alter the miRNA composition of host-derived EVs to promote viral persistence (Chu et al., 2024).

Based on the above findings, we further investigated whether MDV loads its own encoded miRNAs into sEVs. This study reports for the first time that MDV-encoded miRNAs are present in sEVs derived from cell culture supernatants. A total of 10 such miRNAs were detected (**Supplementary Table 2**), among which mdv1-miR-M4-5p_R+2 showed the highest abundance in the MDV infection group, consistent with results from sEVs in the serum of infected chickens. miR-M4-5p is an important oncogenic miRNA of MDV-1 and is homologous to host miR-155 (Zhu et al., 2021). In addition, mdv1-miR-M6-5p_L-2R+1 was highly abundant in the MDV infection group and was upregulated approximately two-fold after chIFN-α treatment, suggesting that it may play an important role in viral evasion of innate immunity. A recent study confirmed that miR-M6-5p promotes MDV latency, proliferation, and tumor formation *in vivo* by directly targeting the histone demethylase KDM2B, thereby epigenetically suppressing the expression of the viral lytic gene pp38 (Zhou et al., 2025). Furthermore, several MDV-encoded miRNAs directly target viral genes and inhibit their expression. For example, miR-M5-3p and miR-M1-5p target and downregulate the immediate-early gene ICP22 (Boumart et al., 2018), miR-M7-5p targets and downregulates the lytic genes ICP4 and ICP27 (Strassheim et al., 2012), and miR-M4-5p targets and downregulates the viral assembly-associated genes UL28 and UL32 (Pegtel et al., 2010). In summary, the discovery of MDV-encoded miRNAs in sEVs, together with the active reprogramming of the overall sEV miRNA profile by MDV, deepens our understanding of the role of viral miRNAs in regulating innate immunity and latent infection.

To decipher the functional logic behind the MDV-reprogrammed sEV miRNA cargo, we performed GO and KEGG enrichment analyzes. GO analysis showed that the predicted targets of these miRNAs are broadly involved in signal transduction, transcription, apoptosis, and proliferation, with an almost equal distribution between positive and negative regulators, suggesting that MDV fine−tunes the host cell state rather than simply activating or suppressing pathways. KEGG analysis further revealed enrichment of the MAPK, Wnt, and endocytosis pathways, with the latter being critical for EV biogenesis and uptake. Condition−specific analyzes (**Supplementary Figure 4**) indicated that MDV predominantly targets metabolic pathways, whereas chIFN−α mainly affects MAPK signaling, and MDV overrides the IFN−α signature during co−treatment. Collectively, these results demonstrate that MDV actively reprograms the sEV miRNA cargo in a condition−dependent manner to suppress antiviral immunity and promote latency. Then we validated that two sEV miRNAs—gga-miR-20a-5p and gga-miR-148a-3p—directly target the 3′UTRs of cGAS and TLR3, respectively, suppressing their expression. This finding reveals a strategic two-pronged inhibition. cGAS has emerged as the predominant cytosolic DNA sensor across most cell types, with its critical role in herpesvirus recognition demonstrated in multiple systems: HSV-1 infection triggers robust cGAS-dependent IFN-I production (Acharya et al., 2025), and murine cytomegalovirus (MCMV) is sensed by cGAS in myeloid cells (Ren et al., 2022). TLR3, in contrast, is an endosomal sensor that recognizes double-stranded RNA, a molecular signature produced during MDV replication, and its activation is a known trigger for IFN-β production (Zou et al., 2017). By simultaneously targeting both sensors, MDV effectively circumvents the redundancy inherent in the host pattern recognition receptor network. Interestingly, this dual-targeting strategy mirrors the convergence of multiple viral immune evasion proteins on the cGAS-STING-TBK1-IRF3 axis observed in other herpesviruses. A comprehensive review by Stempel and colleagues (Stempel et al., 2019) details how herpesviruses have evolved diverse mechanisms to target every step of the DNA sensing pathway. For instance, HSV-1 encodes ICP34.5, which directly targets TBK1 to block signaling (Manivanh et al., 2017), and more recently, the HSV-1 capsid protein UL38 was shown to directly bind STING, blocking cGAMP binding and inhibiting STING-TBK1-IRF3 complex formation (Wang et al., 2025). Similarly, HCMV tegument protein UL82 inhibits STING-mediated signaling by disrupting the MITA-iRhom2-TRAPβ transport complex (Fu et al., 2017). These examples suggest that herpesviruses have convergently evolved to disable this critical pathway at multiple levels, with MDV uniquely employing an EV-miRNA-based mechanism to achieve upstream inhibition of both cGAS and TLR3.

Notably, the loading of these immunosuppressive miRNAs into sEVs suggests a trans-inhibitory mechanism: sEVs released from infected cells can deliver these miRNAs to neighboring, uninfected cells, preemptively suppressing their ability to mount an IFN-I response. This trans-inhibitory mechanism is supported by our labeled sEV tracing experiments, which showed that sEVs derived from infected cells were efficiently internalized by uninfected recipient cells (**Supplementary Figure 2**), confirming that sEVs possess the capacity to transmit signals between cells. This would create a “zone of protection” around infected cells, facilitating viral spread. This mechanism is reminiscent of the bystander immune suppression observed in other viral systems, but with sEVs representing a novel twist on a classic theme. The concept of EVs as vehicles for intercellular immune modulation is increasingly recognized across viral systems. In the context of HIV infection, EVs derived from infected cells have been shown to transfer viral and host miRNAs that induce apoptosis in uninfected CD4^+^ T cells, contributing to T cell depletion (Duette et al., 2018). For HCV, EVs carrying the liver-specific miR-122 can render recipient hepatocytes more permissive to infection (Moghoofei et al., 2021). During SARS-CoV-2 infection, alterations in EV miRNA cargo have been reported to modulate inflammatory responses in recipient cells (Juárez et al., 2025). Our findings extend this emerging paradigm to a cell-associated herpesvirus and demonstrate that MDV not only alters EV miRNA composition but does so in a manner that antagonizes the interferon-induced antiviral state.

Our model of viral hijacking is further supported by evidence of bidirectional miRNA manipulation. Beyond upregulating immunosuppressive miRNAs (Cluster 1), MDV infection also led to the downregulation of specific host miRNAs, such as gga-mir-1672-p5_1ss5GA (Cluster 2). This miRNA is predicted to target the MDV UL36 gene, which encodes a deubiquitinase essential for viral DNA replication (Lin et al., 2020). Downregulation of this miRNA would thus relieve a potential host-imposed block on viral replication, allowing UL36 expression to proceed unhindered. This dual strategy underscores the sophistication of the viral reprogramming of the sEV miRNA landscape. Specifically, MDV simultaneously elevates miRNAs that suppress host immunity while repressing host miRNAs that could directly antagonize viral replication. This finding parallels observations in other herpesvirus systems. During Kaposi’s sarcoma-associated herpesvirus (KSHV) reactivation, the host miR-27 is downregulated, relieving its repression of the viral replication and transcription activator (RTA) and thereby facilitating lytic replication (Quinn and O’Neill, 2014). In EBV infection, the miR-200 family is downregulated, which de-represses viral immediate-early gene expression (Lin et al., 2010). During HSV-1 infection, host miR-101 is downregulated, leading to increased expression of the viral ICP4 protein (Wang et al., 2016). These examples, together with our observation that MDV downregulates a host miRNA predicted to target the essential viral UL36 gene, suggest that bidirectional miRNA manipulation may represent a conserved evolutionary strategy employed by herpesviruses to optimize their replication and persistence. This manipulation involves the simultaneous upregulation of immunosuppressive miRNAs and downregulation of antiviral host miRNAs. The specific miRNAs targeted likely reflect host-specific and virus-specific co-evolutionary pressures, but the overarching principle of exploiting the host miRNA network appears broadly conserved.

The therapeutic potential of targeting EV-mediated communication is increasingly being explored in both cancer and infectious diseases. In oncology, inhibitors of EV biogenesis (such as GW4869) and strategies to deplete circulating EVs have shown promise in preclinical models (Catalano and O’Driscoll, 2020). For viral infections, recent proof-of-concept studies have demonstrated that blocking EV release can reduce viral spread and enhance immune responses (Huang et al., 2018). Our identification of specific miRNAs that mediate MDV immune evasion opens the possibility of using miRNA-based therapeutics as novel antiviral strategies. Examples include antagomirs to neutralize immunosuppressive miRNAs or miRNA mimics to restore antiviral host miRNAs. Furthermore, the sEV miRNA signatures identified in this study may potentially serve as biomarkers for MDV infection or disease progression, analogous to the use of EV-associated miRNAs as diagnostic biomarkers in various cancers and viral diseases (Kumar et al., 2024). Future studies exploring these translational avenues may ultimately lead to new approaches for controlling MDV, an economically significant pathogen, and more broadly inform the development of EV-targeted therapies for other viral infections.

Several limitations of this study should be acknowledged. First, all experiments were conducted *in vitro* using DF-1 cells, an immortalized chicken fibroblast cell line. Although DF-1 cells are a well-established model for MDV research, they may not fully recapitulate the complexity of MDV infection *in vivo*, particularly given that MDV targets lymphocytes during its lytic and latent phases. Second, functional validation of sEV miRNAs was limited to demonstrating direct targeting of the cGAS and TLR3 3′UTRs. Whether these miRNAs are sufficient or necessary for MDV immune evasion in a physiological context remains to be established through functional experiments, such as the use of miRNA mimics and inhibitors. Third, although our labeled sEV tracing experiments confirmed that sEVs can be internalized by recipient cells, the complete chain of evidence demonstrating the transfer of functional sEV miRNAs from infected to uninfected cells and their direct involvement in immune modulation requires further experimental validation. Examples of such approaches include transwell co-culture experiments combined with functional blocking antibodies.

Several promising avenues for future research emerge from this study. First, A comprehensive transcriptomic analysis following miRNA overexpression or inhibition would provide deeper insights into the specificity and global regulatory landscape. *In vivo* validation is essential., developing chicken models to assess the impact of sEV-mediated miRNA transfer on MDV pathogenesis, disease progression, and vaccine efficacy will be a critical next step. Second, the identification of additional sEV miRNAs that target key components of the IFN-I pathway, as well as exploration of other innate immune pathways, may reveal additional layers of viral immune evasion. Third, understanding the mechanism by which MDV selectively packages specific miRNAs into sEVs could reveal novel targets for antiviral intervention. Key questions include whether a viral protein interacts with the miRNA loading machinery or whether viral infection alters the composition of the host Endosomal Sorting Complexes Required for Transport machinery. Fourth, the functional consequences of sEV-mediated miRNA transfer on recipient cells warrant detailed investigation using approaches such as single-cell RNA sequencing. These consequences include changes in recipient cell susceptibility to MDV infection, their ability to mount an IFN-I response, and their potential contribution to MDV-induced lymphomagenesis.

In conclusion, this study elucidates a novel and sophisticated immune evasion paradigm for a cell-associated herpesvirus. We demonstrate that MDV bidirectionally reprograms the sEV miRNA cargo to foster an immunosuppressive extracellular milieu. This is achieved by upregulating specific miRNAs that silence upstream sensors of the IFN-I pathway, namely gga-miR-20a-5p targeting cGAS and gga-miR-148a-3p targeting TLR3, and concurrently downregulating host miRNAs with potential antiviral activity, such as gga-mir-1672-p5_1ss5GA predicted to target the viral UL36 gene. These findings establish “viral co-option of the EV-miRNA axis for systemic immune modulation” as a critical and previously underappreciated dimension of herpesvirus pathogenesis. By revealing how a virus that spreads primarily through cell-to-cell contact can nonetheless exert systemic immunomodulatory effects via sEVs, This work may provide insights into the mechanisms of viral immune evasion and could serve as a reference for the future development of antiviral strategies targeting this intercellular communication pathway.

## Data Availability

The datasets presented in this study can be found in online repositories. The names of the repository/repositories and accession number(s) can be found in the article/**Supplementary Material**.
