# Supplementary figures and images for: Identification of extracellular vesicle microRNAs as potential facilitators of interferon-alpha escape in Marek’s disease virus infection
Source: Front Cell Infect Microbiol. 2026 May 20;16:1796248. doi: 10.3389/fcimb.2026.1796248 (PMC13231281; doi:10.3389/fcimb.2026.1796248)

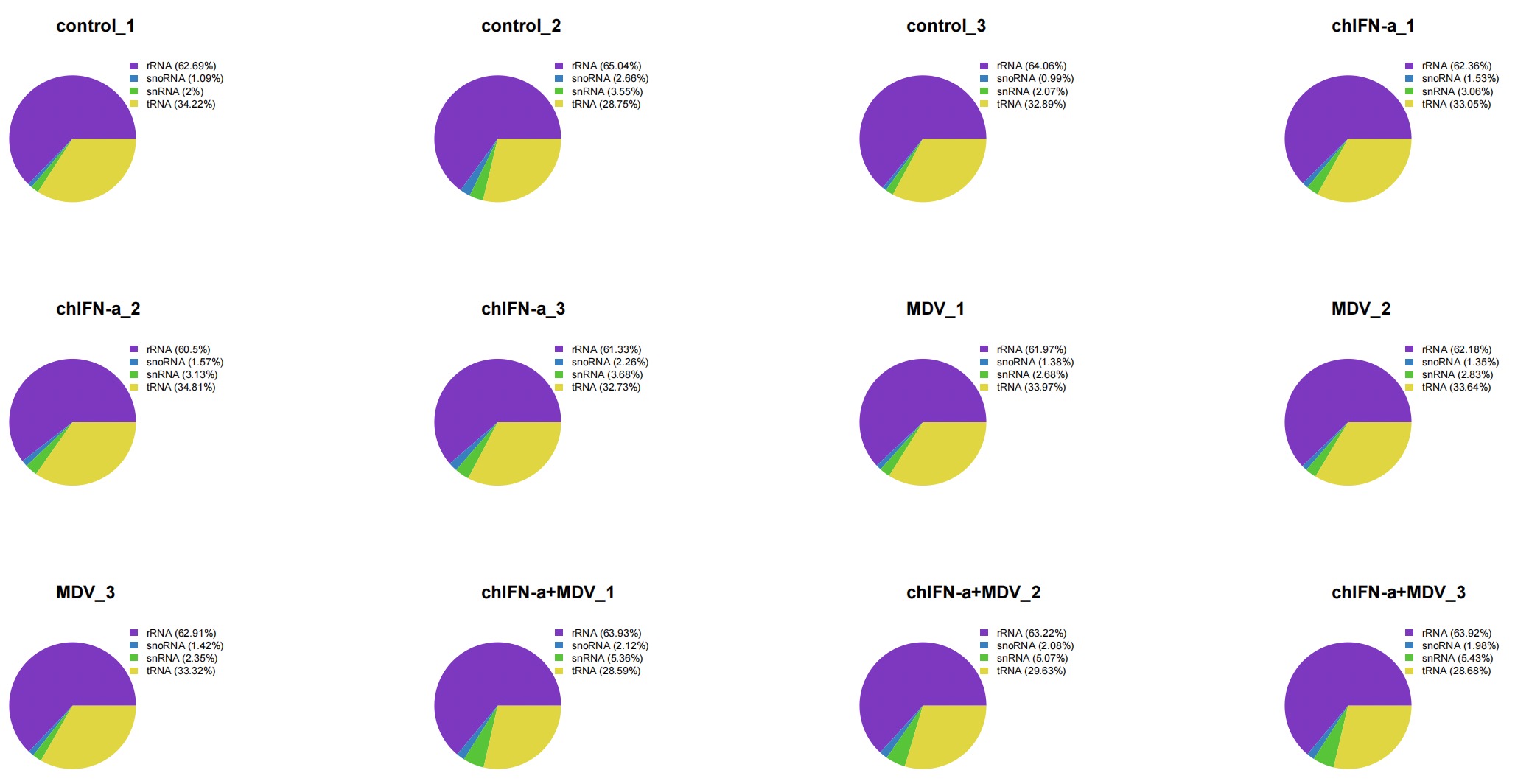

Supplement: Supplementary file 1 [file Image1.jpeg]

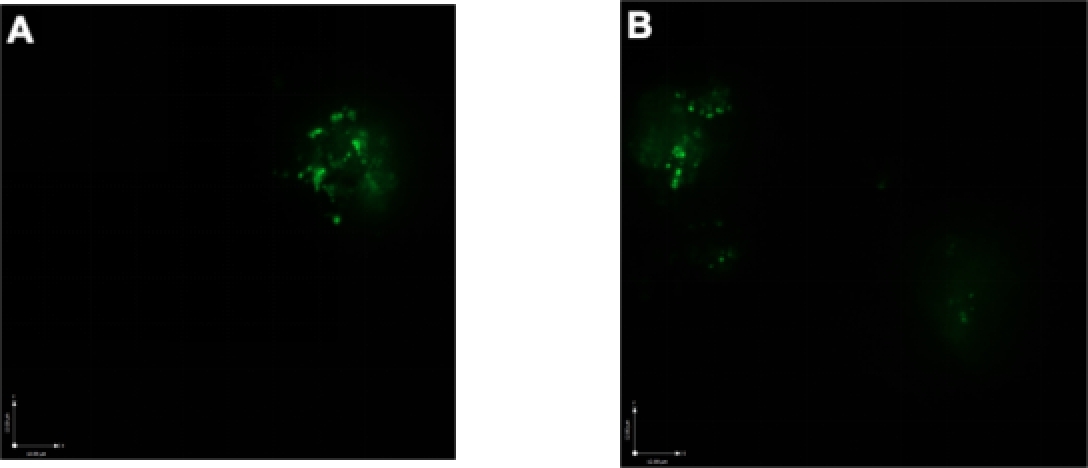

Supplement: Supplementary file 2 [file Image2.jpg]

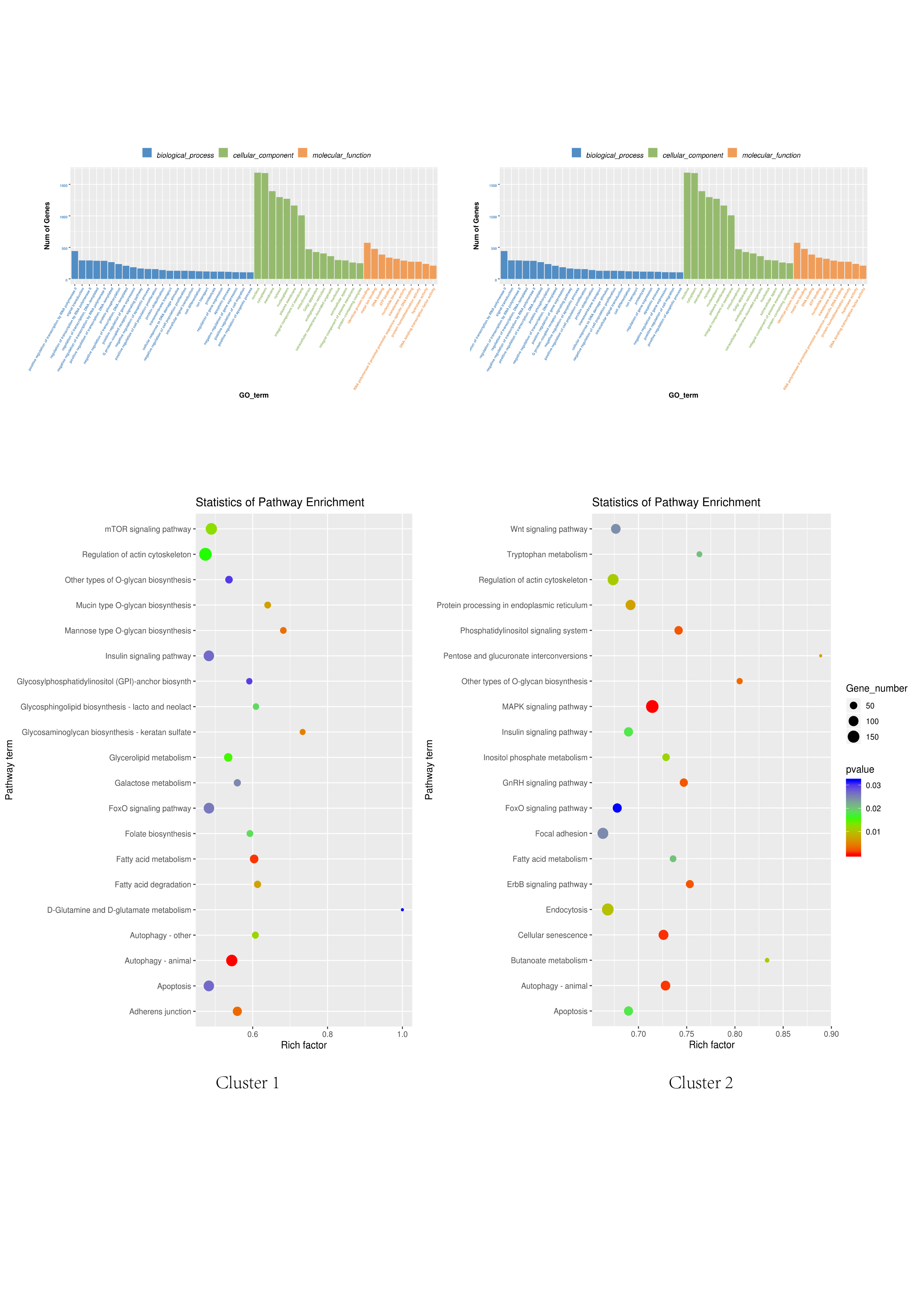

Supplement: Supplementary file 3 [file Image3.tif]

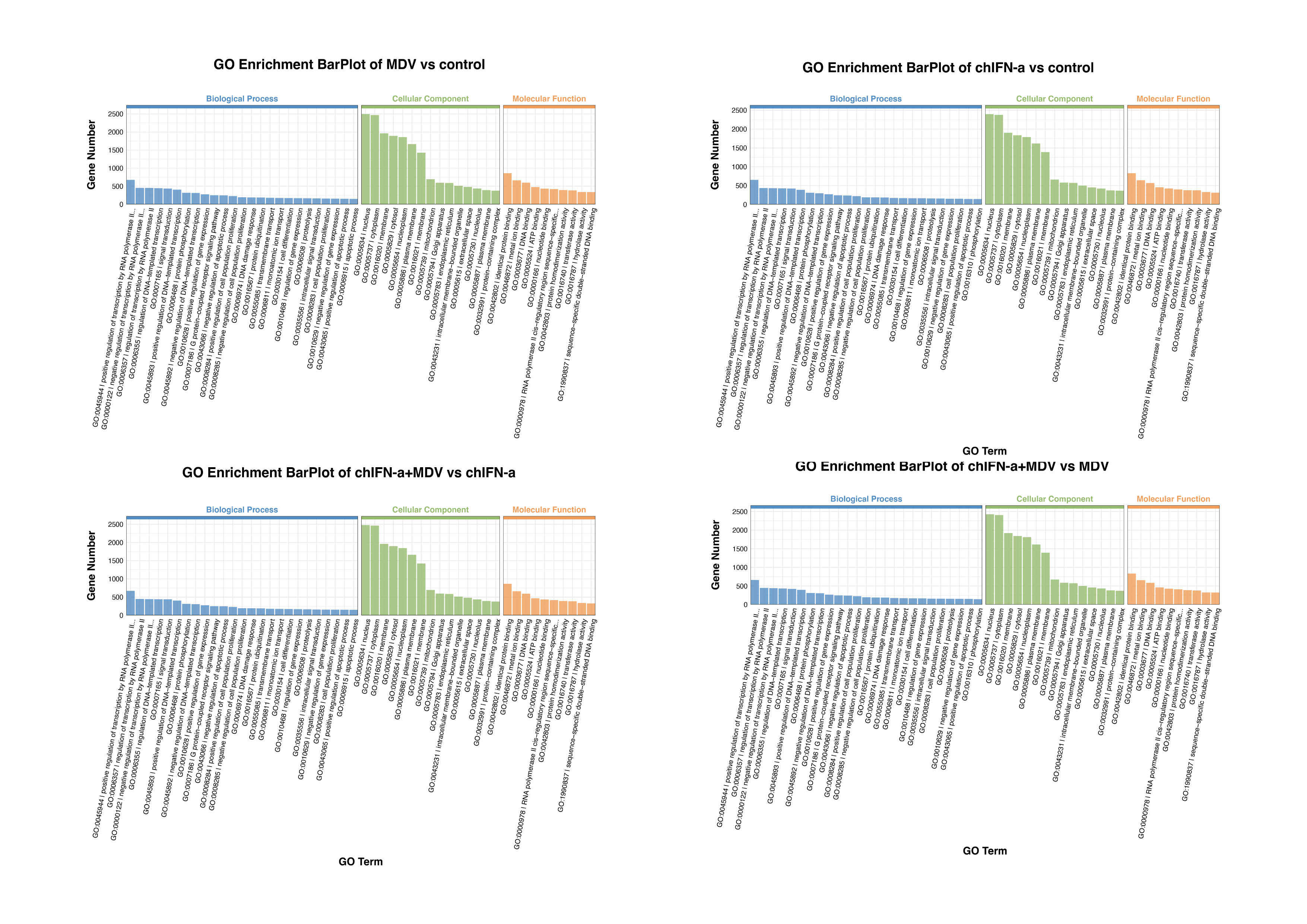

Supplement: Supplementary file 4 [file Image4.tif]

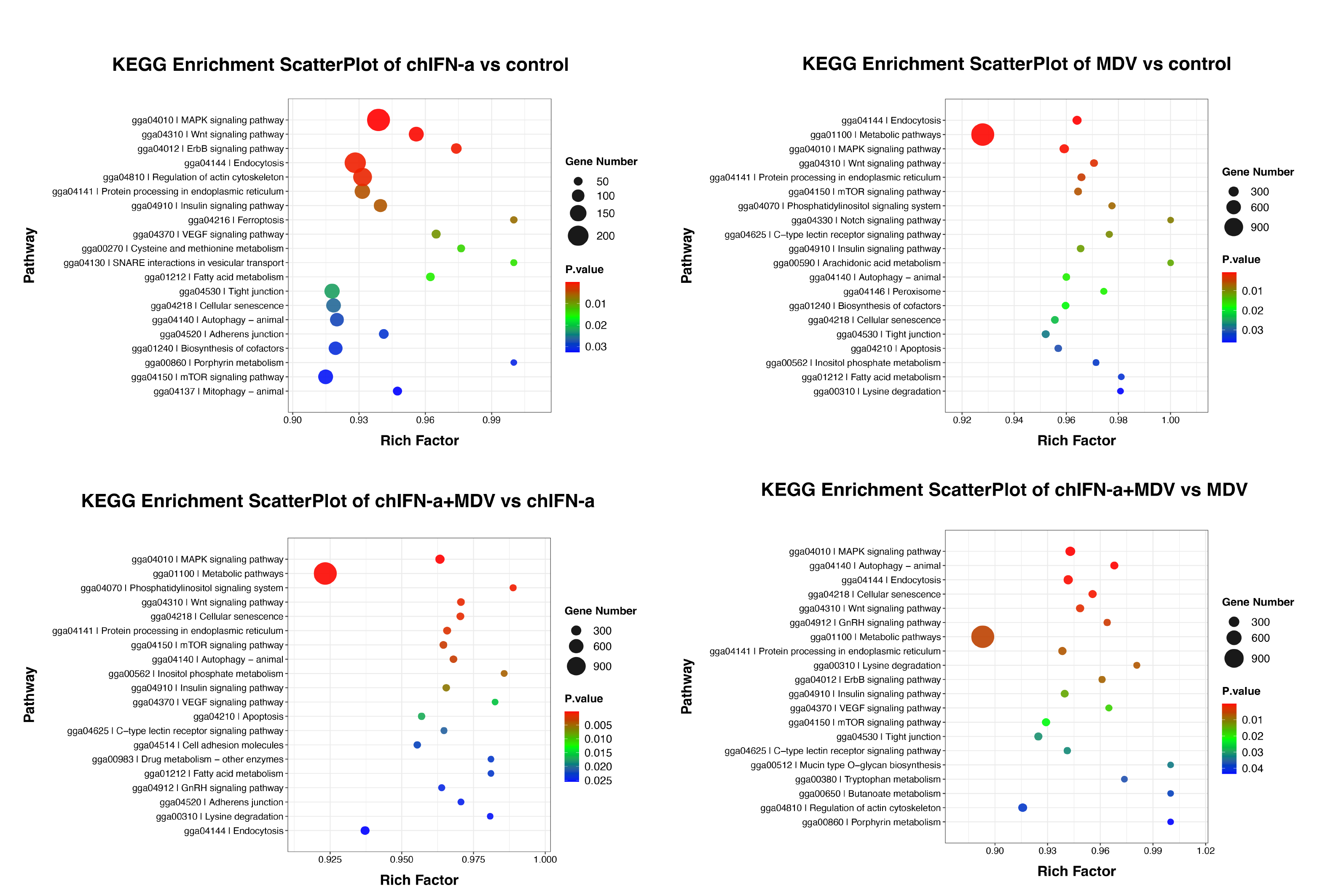

Supplement: Supplementary file 5 [file Image5.tif]
